# Incidence of TPOAb over a 4-year follow-up period: results from the Brazilian Longitudinal Study of Adult Health (ELSA-Brasil)

**DOI:** 10.20945/2359-3997000000422

**Published:** 2021-11-11

**Authors:** Isabela M. Benseñor, Carolina Castro Porto Silva Janovsky, Alessandra C. Goulart, Itamar de Souza Santos, Maria de Fátima Haueisen Sander Diniz, Bianca de Almeida-Pititto, José Augusto Sgarbi, Paulo A. Lotufo

**Affiliations:** 1 Universidade de São Paulo Divisão de Clínica Médica Hospital Universitário São Paulo SP Brasil Centro de Pesquisa Clínica e Epidemiológica, Hospital Universitário, Divisão de Clínica Médica, Universidade de São Paulo, São Paulo, SP, Brasil; 2 Universidade Federal de Minas Gerais Faculdade de Medicina Belo Horizonte MG Brasil Faculdade de Medicina, Universidade Federal de Minas Gerais, Belo Horizonte, MG, Brasil; 3 Universidade de São Paulo Faculdade de Saúde Pública Departamento de Epidemiologia São Paulo SP Brasil Departamento de Epidemiologia, Faculdade de Saúde Pública, Universidade de São Paulo, São Paulo, SP, Brasil; 4 Faculdade de Medicina de Marília Departamento de Medicina Divisão de Endocrinologia e Metabolismo Marília SP Brasil Unidade de Tireoide, Divisão de Endocrinologia e Metabolismo, Departamento de Medicina, Faculdade de Medicina de Marília, Marília, SP, Brasil

**Keywords:** TPOAb, incidence, thyroid function, autoimmune thyroid disease, thyroid disease

## Abstract

**Objective:**

Although some previous data have suggested a high iodine intake in Brazil, the prevalence of antithyroperoxidase antibodies (TPOAb) in the country is compatible with rates from countries with adequate iodine intake. This observation emphasizes the importance of knowing the incidence of TPOAb in Brazil.

**Materials and methods:**

This prospective analysis included euthyroid participants with negative TPOAb at baseline and a thyroid function assessment at a 4-year follow-up. TPOAb was measured by electrochemiluminescence and considered positive when titers were ≥34 IU/mL. TSH and free T4 (FT4) levels were determined by a third-generation immunoenzymatic assay. The incidence of TPOAb is expressed in percentage per year or as a cumulative incidence within the 4-year follow-up period.

**Results:**

Of 8,922 euthyroid participants (mean age 51.1 years; 50.9% women) with a negative TPOAb test at baseline, 130 presented incident TPOAb at the 4-year follow-up, yielding an annual incidence of TPOAb of 0.38%/year (95% confidence interval [95% CI], 0.37-0.39%/year) and a cumulative incidence over 4 years of 1.46% (95% CI, 1.21-1.71%). In men, the annual incidence was 0.32% (95% CI, 0.31-0.33%), and the cumulative incidence over 4 years was 1.23% (95% CI, 0.90-1.56%). In women, the annual incidence was 0.43%/year (95% CI, 0.42-0.44%/year) and the cumulative incidence over 4 years was 1.67% (95% CI, 1.30-2.04%). The only factor associated with incident TPOAb was the occurrence of thyroid diseases at follow-up. No differences in TPOAb incidence were detected across ELSA-Brasil research centers.

**Conclusion:**

Based on the results of this study, the incidence of TPOAb per year and at a 4-year follow-up period are compatible with those of a country with adequate iodine intake.

## INTRODUCTION

Thyroid disease is a global health problem with the potential of substantially impacting the health of the population. Overt and subclinical hypothyroidism due to autoimmune thyroiditis are the most common types of thyroid dysfunction in iodine-sufficient populations (1-3). In this context, the most important aspect for the diagnosis of autoimmune thyroid diseases is the presence of circulating antithyroperoxidase antibodies (TPOAb) (3-6).

Thyroid diseases are very common in Brazil (7-9), a country considered to have one of the highest prevalences of hypothyroidism and hyperthyroidism worldwide (10). Although goiter was a common presentation of thyroid diseases in the past, the prevalence of this condition has decreased after compulsory salt iodination in the second half of the 20^th^ century. A previous analysis with data from the Brazilian Longitudinal Study of Adult Health (ELSA-Brasil) has shown that the prevalence of TPOAb positivity in Brazil was aligned with that of iodine-sufficient areas (11), and was higher in women compared with men, with a women:men ratio of 2.2. Additionally, 60% of the individuals with positive TPOAb were white. The presence of positive TPOAb in women was associated with the entire spectrum of thyroid dysfunction, while in men, it was related only to the occurrence of overt thyroid disease (11). To the best of our knowledge, no large studies have been published on the incidence of autoimmune thyroid diseases in Brazil.

The present study aimed to analyze the incidence of autoimmune thyroiditis considering all participants who had negative TPOAb levels and no thyroid dysfunction at baseline (2008-2010) and were followed up during 4 years (2012-2014). The incidence of TPOAb along the Brazilian Longitudinal Study of Adult Health (ELSA-Brasil) is presented according to the baseline sociodemographic and clinical characteristics of the participants.

## MATERIALS AND METHODS

This was a prospective analysis based on a 4-year follow-up period of the ELSA-Brasil. The design and cohort profile of the ELSA-Brasil study have been previously published elsewhere (12-14). Briefly, the ELSA-Brasil is a multicenter cohort study of 15,105 active and retired civil servant employees aged between 35 and 74 years at baseline and from six cities in Brazil (Salvador, Vitória, Belo Horizonte, Rio de Janeiro, São Paulo, and Porto Alegre). Information on sociodemographic data, personal and family history of previous diseases, lifestyle risk factors, mental health, cognitive status, and occupational exposure was assessed at baseline from 2008 to 2010 and after 4 years from 2012 to 2014.

The institutional ethics committee approved the protocol of the study, and written consent was obtained from all study participants (Plataforma Brasil CAAE number 08109612.7.1001.0076).

### Participants

**Figure 1:**
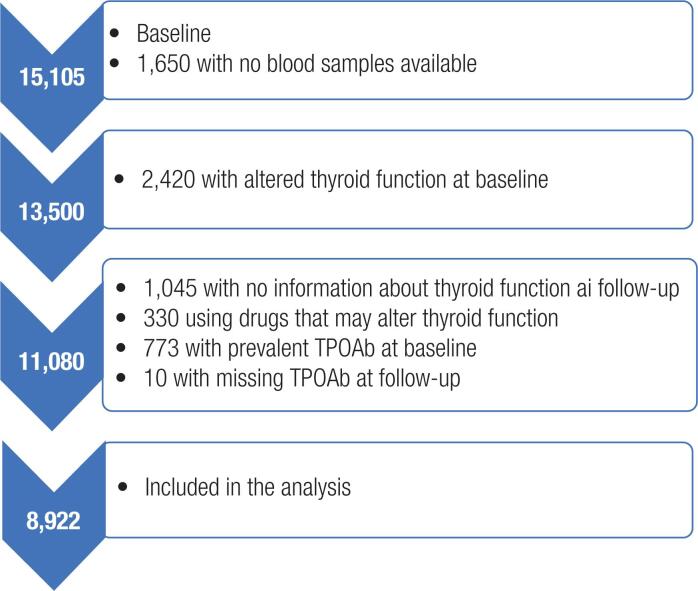
Participants included in the analysis.

Thyroid function tests were assessed in frozen samples stored in nitrogen. The present analysis included all participants with stored baseline samples available at the moment of the analysis (n = 13,500). No differences regarding sociodemographic or clinical characteristics were detected among participants with and without frozen samples. After exclusion of all participants with abnormal thyroid function (n = 2,420) only participants with normal thyroid function remained in the study (n = 11,080). Of 11,080 participants with normal thyroid function at baseline, we excluded 1,045 with missing information about thyroid function at follow-up, 330 who were using medications that could interfere with thyroid function assessment (15,16), 773 with positive TPOAb at baseline, and 10 with missing information about TPOAb at the follow-up assessment. After these exclusions, the final analysis comprised 8,922 participants (Figure 1).

### Thyroid function

Serum for biochemistry and hormone tests was obtained from venous blood samples drawn after an overnight fast and centrifuged at 2,500 *g* for 15 minutes. Serum TPOAb was measured by electrochemiluminescence (Roche Diagnostics, Mannheim, Germany) and was considered positive when ≥34 IU/mL. Serum levels of TSH and FT4 were determined using a third-generation immunoenzymatic assay (Roche, USA). Serum free triiodothyronine was measured by electrochemiluminescence immunoassay. Thyroid dysfunction was defined by serum TSH and FT4 levels or by the routine use of thyroid hormones (levothyroxine) or antithyroid medications such as propylthiouracil or methimazole. Reference ranges were 0.4-4.0 mIU/L for normal TSH levels and 0.93-1.7 ng/dL for normal FT4 levels. Based on their TSH and FT4 levels and use of medications to treat thyroid disorders, the participants were classified into one of the following categories of thyroid function: overt hyperthyroidism (low TSH, high FT4, or use of medications to treat hyperthyroidism), subclinical hyperthyroidism (low TSH, normal FT4, and no use of thyroid medication), euthyroidism (normal TSH with no use of thyroid medication), subclinical hypothyroidism (high TSH, normal FT4, and no use of thyroid medication), and overt hypothyroidism (high TSH, low FT4, or use of levothyroxine) (17).

### Other variables

Fasting plasma glucose was measured using a hexokinase method. The study questionnaire addressed sociodemographic factors including age (as continuous and categorical [age strata] values, *i.e.*, 35-44, 45-54, 55-64, and 65-74 years), sex, educational level (less than high school, high school and some college, or complete college or more), self-reported race/skin color (white, mixed color, black, Asian descendant, or Brazilian indigenous), smoking status (never, past, or current), alcohol use (never, past, or current), hypertension (yes or no), diabetes (yes or no), and dyslipidemia (yes or no). All participants were asked about their use of prescription and nonprescription medications within 2 weeks from their visits to the research center (18).

Weight was measured with the participant wearing light clothes. Body mass index (BMI) was calculated as weight divided by squared height (kg/m^2^). Waist circumference was measured at the midpoint between the lower edge of the last rib and the iliac crest at the midaxillary line. Blood pressure was measured after a 5-minute rest; three measurements were taken at 1-minute intervals between each measurement, and the mean of the second and third measurement was considered as the participant’s casual blood pressure level. Participants who reported the use of medication to treat hypertension or had a systolic blood pressure ≥140 mmHg or a diastolic blood pressure ≥90 mmHg were considered to have hypertension. Those participants who reported a previous medical history of diabetes mellitus or use of medication to treat this condition, presented a fasting plasma glucose ≥126 mg/dL (measured only once) or a 2-hour plasma glucose ≥200 mg/dL after 75 g of oral anhydrous glucose, or had a level of glycated hemoglobin (HbA1c) level ≥6.5% were considered to have diabetes mellitus. Dyslipidemia was defined as an LDL-cholesterol level >130 mg/dL or the use of lowering cholesterol drugs.

### Statistical analysis

Categorical variables were reported as proportions and compared using the chi-square test. Continuous parametric variables were reported as means (standard deviations [SD]) and compared using analysis of variance (ANOVA), while continuous nonparametric variables were reported as medians (interquartile ranges) and compared using nonparametric tests (Mann-Whitney or Kruskal-Wallis test, as appropriate). The variable “TPOAb” was log-transformed and compared using ANOVA across all ELSA-Brasil research centers, then back-transformed to their original values. The incidence of TPOAb is expressed in percentage/year or as a cumulative incidence over the 4-year follow-up period. A logistic regression model was constructed to analyze the association between the TPOAb incidence and thyroid function (using euthyroidism as a reference), and models were presented after adjustment for age, sex, education level, and race. All analyses were performed using the Statistical Package for Social Sciences version 25.0 (IBM SPSS Statistics). A p value < 0.05 was considered significant.

## RESULTS

Of the 8,922 euthyroid participants (mean age 51.1 years; 50.9% women) who presented negative TPOAb at baseline, only 130 presented incident TPOAb at follow-up, yielding an annual incidence of TPOAb of 0.38%/year (95% confidence interval [95% CI], 0.37-0.39%/year) and a cumulative incidence over 4 years of 1.46% (95% CI, 1.21-1.71%). In men, the annual incidence was 0.32% (95% CI, 0.31-0.33%), and the cumulative incidence over 4 years was 1.23% (95% CI, 0.9-1.56%). In women, the annual incidence was 0.43%/year (95% CI, 0.42-0.44%/year) and the cumulative incidence over 4 years was 1.67% (95% CI, 1.30-2.04%) (Figure 1). The women:men ratio was 1.36.

No differences in TPOAb incidence were detected across the ELSA-Brasil research centers, but the median values of incident TPOAb were higher in Belo Horizonte compared with the other centers. The median TPOAb titers per ELSA-Brasil research center, from lowest to highest, were as follows: São Paulo, 49.7 IU/mL (36.6-81 IU/mL); Rio de Janeiro, 56.5 IU/mL (37.5-110.3 IU/mL); Salvador, 57.5 IU/mL (38-90 IU/mL), Porto Alegre, 58.7 IU/mL (37.3-102.5 IU/mL); and Belo Horizonte, 77.3 IU/mL (39.7-77.3 IU/mL). Since only 2 cases of incident TPOAb were identified in Vitória, this research center was excluded from the subanalysis comparing the median values of incident TPOAb across research centers.

Table 1 describes the baseline (2008-2010) sociodemographic and clinical characteristics of the ELSA-Brasil participants according to the occurrence or not of incident TPOAb at the 4-year follow-up assessment (2012-2014). The participants with incident TPOAb were younger and more frequently women compared with the participants with no incident TPOAb, although these differences were not statistically significant (p = 0.06 for age and p = 0.08 for sex). No differences in incident TPOAb were found in relation to age strata, self-declared race, education level, or status of current smoking or alcohol intake.

**Table 1:** General characteristics of the participants according to the occurrence or not of incident antithyroperoxidase antibodies (TPOAb) at follow-up

		Incident TPOAb		p
		Yes N = 130	No N = 8,792	
Sex (%)				0.08
	Women	76 (58.5)	4,467 (50.8)	
	Men	54 (41.5)	4,325 (49.2)	
Age (years) (mean, standard deviation)		49.7 (8.2)	51.2 (8.9)	0.06
Age strata (years)				0.42
	35-44	38 (29.2)	2200 (25)	
	45-54	56 (43.1)	3581 (40.7)	
	55-64	28 (21.5)	2259 (25.7)	
	65-74	8 (6.2)	752 (8.6)	
Body mass index (BMI) kg/m^2^		27.3 (5.2)	26.8 (4.6)	0.25
Waist circumference (cm)		90.9 (12.8)	90.8 (12.5)	0.98
State capitals				0.45
	Salvador	18 (13.8)	1,081 (12.3)	
	Vitória	2 (1.5)	395 (4.5)	
	Belo Horizonte	27 (20.8)	1,784 (20.3)	
	Rio de Janeiro	16 (12.3)	1,076 (12.2)	
	São Paulo	13 (10)	3,258 (13.6)	
	Porto Alegre	54 (41.5)	1,198 (37.5)	
Self-declared race				0.41
	White	55 (42.6)	4,459 (51.3)	
	Mixed color	42 (32.6)	2,437 (28.1)	
	Black	26 (20.2)	1,458 (16.8)	
	Asian descendant	4 (3.1)	238 (2.7)	
	Brazilian indigenous	2 (1.6)	95 (1.1)	
Education level				0.58
	Less than high school	15 (11.5)	1,053 (12)	
	High school and some college	52 (40)	3,132 (35.6)	
	At least complete college	63 (48.5)	4,607 (52.4)	
Smoking				0.35
	Never	66 (50.8)	5,016 (57.1)	
	Past	43 (33.1)	2,576 (29.3)	
	Current	21 (16.2)	1,199 (13.6)	
Alcohol intake				0.46
	Never	10 (7.7)	890 (10.1)	
	Past	29 (22.3)	1,663 (18.9)	
	Current	91 (70)	6,231 (70.9)	
Physical Activity				0.34
	Active	31 (24.2)	2,197 (25.3)	
	Insufficiently active	11 (8.6)	1,089 (12.6)	
	Inactive	86 (67.2)	5,384 (62.1)	
Hypertension (%) mmHg		41 (31.5)	2,902 (33)	0.72
Diabetes (%)		21 (16.2)	1,582 (18)	0.59
Dyslipidemia		70 (53.8)	5,036 (57.3)	0.43
Current thyroid function at visit 2 (%)				<0.0001
	Overt hyperthyroidism	2 (1.5)	10 (0.1)	
	Subclinical hyperthyroidism	4 (3.1)	36 (0.4)	
	Euthyroidism	115 (88.5)	8,306 (94.5)	
	Subclinical hypothyroidism	4 (3.1)	315 (3.6)	
	Overt hypothyroidism	5 (3.8)	125 (1.4)	
Thyroid-stimulating hormone (TSH) mIU/mL		1.98 1.39-2.66	1.81 1.30-2.48	0.07
Free thyroxine (FT4) ng/dL		1.16 1.04-1.32	1.18 1.08-1.28	0.42
Free triiodothyronine (FT3) pg/mL		0.31 0.28-0.34	0.31 0.28-0.33	0.50
TPOAb (IU/mL)		55.1 37.6-92.6	10.75 8.53-13.71	<0.0001

**Figure 2:**
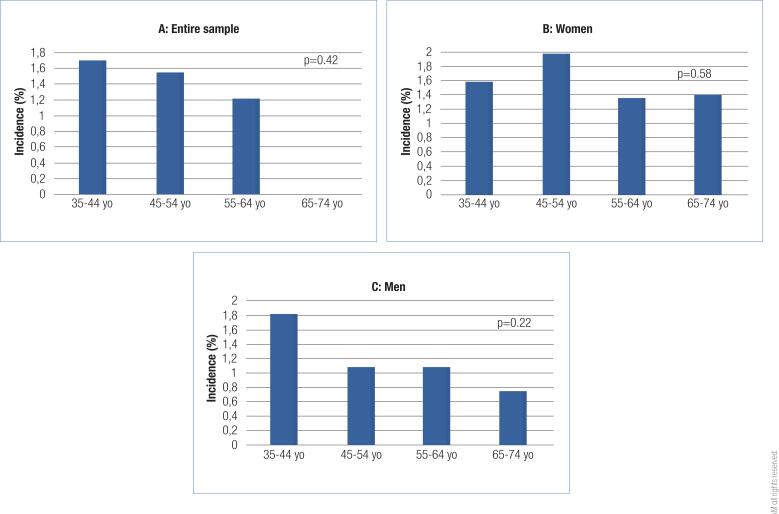
Cumulative incidence of antithyroperoxidase antibodies (TPOAb) according to age strata in: (**A**) Entire sample, (**B**) Mean and (**C**) Women.

Figure 2 shows the cumulative incidence of TPOAb (percentage in a 4-year period) according to age strata and sex. There was a decline in TPOAb incidence by age strata in the entire sample and in women and men, but the differences were not statistically significant (p = 0.42, p = 0.58, and p = 0.22, respectively).

The only factor associated with incident TPOAb was thyroid function at follow-up (p < 0.0001). Most incident cases occurred in participants with normal thyroid function at follow-up (88.5%), while the remaining cases were distributed according to incident thyroid diseases at follow-up as overt hyperthyroidism (1.5%), subclinical hyperthyroidism (3.1%), subclinical hypothyroidism (3.1%), and overt hypothyroidism (3.8%). In a logistic regression model adjusted by sex, age, and race and using euthyroidism as a reference, incident TPOAb was associated with overt hyperthyroidism (odds ratio [OR] 13.9; 95% CI, 3.0-64.9), subclinical hyperthyroidism (OR 8.4; 95% CI, 2.9-24.3), and overt hypothyroidism (OR 3.0; 95% CI, 1.2–7.5) but not with subclinical hypothyroidism (OR 1.0; 95% CI, 0.4-2.7).

**Figure 3:**
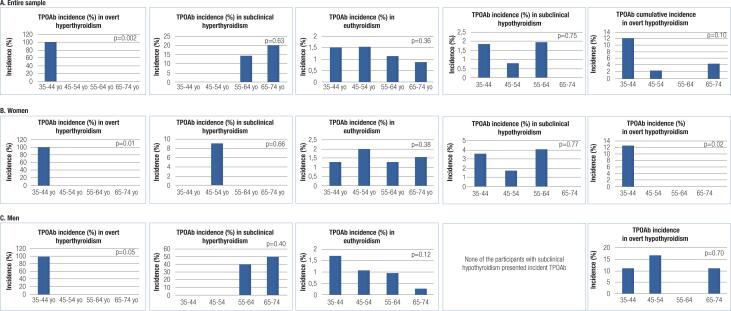
Distribution of incidence of anthyroperoxidase antibodies (TPOAb) according to thyroid function at follow-up, age strata an sex (A: Entire sample; B: Women; C: Men. (yo – years of age).

Figure 3 shows the distribution of incident TPOAb according to the presence of thyroid diseases and age strata at follow-up for the entire sample and according to sex. All cases of overt hyperthyroidism in the entire sample (p = 0.002) and in women (p = 0.01) and men (with borderline significance, p = 0.05) are concentrated in the age strata of 35-44 years. Incident TPOAb was only associated with overt hypothyroidism in women in the younger age strata (35-44 years).

## DISCUSSION

The results of the present study showed an annual incidence of TPOAb over a 4-year follow-up period that was compatible with previous studies in iodine-sufficient populations (3,5,6). The only factor associated with incident TPOAb was the presence at follow-up of thyroid dysfunction, except for subclinical hypothyroidism. The women:men ratio of incident TPOAb was 1.36. Incident TPOAb in overt hyperthyroidism was concentrated in the younger age strata in the entire sample and in women, but this observation was only borderline significant in men. Additionally, incident TPOAb was only associated with overt hypothyroidism in women in the younger age strata (35-54 years). We observed no differences in the incidence of TPOAb across ELSA-Brasil research centers, although the Belo Horizonte site had higher levels of incident TPOAb compared with the other centers (p < 0.0001). Incident TPOAb was not associated with age when analyzed as a continuous variable or with sex.

The incidence of TPOAb was defined in percentage per year or cumulative incidence over a 4-year period. Since thyroid function was only measured at baseline and at the 4-year follow-up period and no information was available about thyroid function during the 4-year interval, we opted for not presenting the results in persons-year since the time interval was around 4 years for all participants.

Although several studies have evaluated the prevalence of TPOAb, only a small number of studies have evaluated the incidence of TPOAb or other antithyroid antibodies. To the best of our knowledge, this is the first large study investigating the incidence of TPOAb in Brazil. During a 20-year follow-up in the Whickham Study, 17.3% of the women and 6.6% of the men developed incident antithyroid antibodies (antithyroglobulin, antithyroid cytoplasmic antibodies, and antimicrosomal antibodies) (19). In the recent Tehran Thyroid Study, in which a sample of 2,171 men and 2,849 women with negative TPOAb at baseline was evaluated after a median follow-up of 9.1 years, there were 77 cases of incident TPOAb in men and 223 in women, corresponding to an incidence of new cases of 0.66%/year (0.39%/year in men and 0.86%/year in women) (6). Additionally, Li and cols. have reported a 5-year incidence of TPOAb of around 2.8% (20). Compared with these studies, the incidences per year and over a 4-year follow-up were lower in our study (6,18). We were unable to compare our data with those from the Whickham Study since the latter measured antimicrosomal antibodies. The women:men ratio of 2.2 in a study by Amouzegar and cols. was higher than the corresponding ratio found in our sample (1.36). This finding in our study is aligned with the results from a previous Brazilian population-based study that found a prevalence of TPOAb of 10.7% in women and 9.2% in men, with a women:men ratio of 1.2. (8) This type of comparison across studies is very challenging since TPOAb may have been measured using different techniques, while in older studies, other types of antibodies were tested, as in the case of the Whickham Study (21). Additionally, the way that incidence has been reported in each study – percentage per year, cumulative incidence over a period, or persons-year – are additional challenges for establishing direct comparisons between the studies. Other sociodemographic risk factors and environmental characteristics related to iodine and intake of other micronutrients or even genetic differences among the study populations may also interfere with incidences obtained from different populations.

We found no association between incident TPOAb and sex or age. The Wickham Study found higher levels of antithyroid antibodies (21) in women compared with men and an association of positive antithyroid antibodies with age in women but not in men. Data from the National Health and Nutrition Examination Survey (NHANES III) showed an increasing prevalence of TPOAb with age in both men and women (22). In the present analysis, TPOAb levels increased only 4% from the first (35-44 years) to the fourth (65-74 years) age strata (11). Another possible explanation for our results is that, due to the small number of cases of incident TPOAb, the study may not have had enough power to detect differences in incidence according to age. The ELSA-Brasil cohort comprises middle-aged men and women, and most of the cohort at baseline had a mean age below 55 years, which may also help explain our results. However, our results are aligned with those of a previous population-based study in older individuals in Brazil that also showed no differences in the prevalence of TPOAb according to age and sex (8). In the study by Amouzegar and cols., performed in another low- and middle-income country, incident TPOAb was concentrated among younger individuals compared with middle-aged and older participants (6). In China, incident TPOAb had no association with sex (18). These results are more similar to ours and are probably explained by a higher early mortality rate in low- and middle-income countries compared with high-income ones. It was only when we analyzed incident TPOAb in relation to overt thyroid diseases at follow-up that we found an association in the lower age strata for overt hyperthyroidism (in the entire cohort and in women, with a borderline significance in men) and for overt hypothyroidism in women. An important aspect related to thyroid diseases in the present analysis was that despite the increased frequency of incident TPOAb in women compared with men, the women:men ratio was low not only for TPOAb incidence but also for overt hyperthyroidism and hypothyroidism. In our sample, the women:men ratio for thyroid diseases was 1.36, which is in accord with a previous study in which the reported ratio was 1.2 (8) but different from the ratios reported in other classical studies (19,23) on thyroid diseases.

Our study found no association between subclinical hypothyroidism and positive TPOAb. The most likely explanations for this observation are that the incidence of TPOAb in the sample was not high, that some imbalance could have occurred across thyroid function categories, and that this finding could have occurred by chance. The small number of individuals with positive TPOAb among the participants with subclinical hypothyroidism could explain the lack of significance of this association. Further, the short follow-up (4 years) might also explain the low numbers of incident cases.

The World Health Organization (WHO) considers Brazil to be a country with sufficient iodine intake (24). However, results of the more recent PNAISAL survey have classified Brazil as a country with a more than adequate iodine intake (25). A recent meta-analysis of Brazilian studies has shown heterogeneous data with most of the studies located in the South and Southeast regions and without enough information from other areas of the country where insufficient iodine intake is still possible (26). Our previous analysis about the prevalence of TPOAb using data from the ELSA-Brasil cohort has reported a higher prevalence of TPOAb in Belo Horizonte. In this present analysis of incidence, we found no differences across ELSA-Brasil research centers (11). Excessive iodine intake is associated with thyroid autoimmunity (1-3) and a likely effect on TPOAb levels, but such an effect was not observed in our sample. However, considering the results of the PNAISAL survey, increase in TPOAb titers in samples from Brazil is possible in the near future.

We found no association of incident TPOAb and self-declared race in our sample. Some studies have suggested that Hashimoto’s thyroiditis is associated with white race (9,27) and that Graves’ disease is associated with black race (27,28) In the present analysis, we found no association between race and incident TPOAb. Although smoking is associated with decreased TPOAb levels (29,30), we found no association between smoking status and incident TPOAb in our sample. The small prevalence of current smoking in our sample may have influenced this result.

The present study has some strengths. The ELSA-Brasil is a multicenter cohort study in which repeated measurements of thyroid function allowed for a prospective analysis. The data were collected under strict quality control, and serum TSH, FT4, and TPOAb levels were measured using the same kits at both timepoints. The cohort includes a sample of apparently healthy individuals, and the blood samples were only taken if the participants had no clinical symptoms during their visits to the research center. In the presence of symptoms (*e.g.*, fever), the blood collection was postponed. The study also has some limitations. Levels of thyroid hormones and TPOAb were only measured once in both visits. Since the incidence of TPOAb was not high in our sample, some imbalance across thyroid function categories was possible and caused by chance. This is the case of the absence of association of TPOAb and subclinical hypothyroidism, which may be explained by the low number of individuals with incident TPOAb among participants with subclinical hypothyroidism. The median follow-up of around 4 years was short, and there were only a small number of incident cases in the sample. Also, we have no information about antithyroglobulin antibodies – which is a relevant marker of iodine intake and may be related to TSH levels and Hashimoto’s thyroiditis – or about genetic variants at this moment of the study, which limits our ability to evaluate the risk factors associated with autoimmune thyroid diseases. Lastly, the study included only individuals aged 35-74 years.

In conclusion, our findings on incident TPOAb are aligned with those of a country with sufficient iodine intake. Still, monitoring the incidence of TPOAb over the next few years will be fundamental since the changes in iodine intake in Brazil – from “sufficient” to “more than adequate” – may have an effect on thyroid autoimmunity and TPOAb levels in the country in the near future.
